# Environmental Health Research in Africa: Important Progress and Promising Opportunities

**DOI:** 10.3389/fgene.2019.01166

**Published:** 2020-01-16

**Authors:** Bonnie R. Joubert, Stacey N. Mantooth, Kimberly A. McAllister

**Affiliations:** ^1^National Institute of Environmental Health Sciences, National Institutes of Health, Durham, NC, United States; ^2^VISTA Technology Services, Durham, NC, United States

**Keywords:** G x E, Africa, environmental, pesticides, metals, mold, air pollution

## Abstract

The World Health Organization in 2016 estimated that over 20% of the global disease burden and deaths were attributed to modifiable environmental factors. However, data clearly characterizing the impact of environmental exposures and health endpoints in African populations is limited. To describe recent progress and identify important research gaps, we reviewed literature on environmental health research in African populations over the last decade, as well as research incorporating both genomic and environmental factors. We queried PubMed for peer-reviewed research articles, reviews, or books examining environmental exposures and health outcomes in human populations in Africa. Searches utilized medical subheading (MeSH) terms for environmental exposure categories listed in the March 2018 US National Report on Human Exposure to Environmental Chemicals, which includes chemicals with worldwide distributions. Our search strategy retrieved 540 relevant publications, with studies evaluating health impacts of ambient air pollution (n=105), indoor air pollution (n = 166), heavy metals (n = 130), pesticides (n = 95), dietary mold (n = 61), indoor mold (n = 9), per- and polyfluoroalkyl substances (PFASs, n = 0), electronic waste (n = 9), environmental phenols (n = 4), flame retardants (n = 8), and phthalates (n = 3), where publications could belong to more than one exposure category. Only 23 publications characterized both environmental and genomic risk factors. Cardiovascular and respiratory health endpoints impacted by air pollution were comparable to observations in other countries. Air pollution exposures unique to Africa and some other resource limited settings were dust and specific occupational exposures. Literature describing harmful health effects of metals, pesticides, and dietary mold represented a context unique to Africa. Studies of exposures to phthalates, PFASs, phenols, and flame retardants were very limited. These results underscore the need for further focus on current and emerging environmental and chemical health risks as well as better integration of genomic and environmental factors in African research studies. Environmental exposures with distinct routes of exposure, unique co-exposures and co-morbidities, combined with the extensive genomic diversity in Africa may lead to the identification of novel mechanisms underlying complex disease and promising potential for translation to global public health.

## Introduction

A global assessment by the World Health Organization (WHO) in 2016 estimated that 24% of the global disease burden and 23% of all deaths were attributed to modifiable environmental factors, including physical, chemical, and biological hazards to human health (Prüss-Ustün et al., 2016). The highest number of deaths per capita attributable to the environment was reported for sub-Saharan Africa, primarily reflecting infectious diseases, but also noncommunicable diseases and injuries. Disease burden was highest (36%) among children. In modern Africa, there has been rapid industrial development in the absence of health and environmental safety guidelines that parallel those in the United States, Canada, or Europe (Organization, 2017). Heavy metals, pesticides, air pollution, water contaminants, and waste represent hazardous exposures increasing in Africa (Nweke and Sanders, 2009), but with limited research attention on the implications for human health. Many chemicals that pose health risks to exposed populations in Africa and around the world are known to be endocrine disrupting chemicals (EDCs). A meeting of scientists around this issue took place in South Africa in 2015, leading to a “call to action” to utilize available scientific knowledge to address the impact of EDCs on human as well as wildlife health in Africa (Bornman et al., 2017). This meeting report also called for a shift from reaction to prevention, with utilization of existing datasets, increased biomonitoring, and surveillance of environmental chemicals, as well as further research including the support of longitudinal studies (Bornman et al., 2017).

Often in parallel to environmental health research, genomic research related to The Human Genome Project has advanced our understanding of disease susceptibility with enormous productivity and ongoing promise. Initial research in genomics had limited participation from African study populations, despite the important genomic diversity represented by African populations. However, huge efforts to address this limitation took place in the last decade resulting in an ongoing genomic research revolution in Africa (Consortium et al., 2014). Much of that effort was enabled by investments from the African Society of Human Genetics, National Institutes of Health (NIH), and the Wellcome Trust through the Human Heredity and Health in Africa (H3Africa) consortium (www.h3africa.org). The H3Africa consortium began in June 2010 to support genomic and epidemiological research led by African scientists (Consortium et al., 2014). Genomic research in Africa is not limited to the bounds of this consortium, but it represents a research infrastructure that enables innovative science. For example, studies covering common diseases such as cardiovascular (Owolabi et al., 2014), neurological (Akinyemi et al., 2016), respiratory (Zar et al., 2016a; Zar et al., 2016b), kidney (Osafo et al., 2015), and other non-communicable diseases are represented in this consortium. Developments in pharmacogenomics (Warnich et al., 2011) and the human microbiome (Adebamowo et al., 2017) are also underway, and many studies incorporate information about HIV, malaria, tuberculosis, and other common infections in Africa. The H3Africa consortium also promotes opportunities for training in bioinformatics (Adoga et al., 2014; Oluwagbemi et al., 2014; Mulder et al., 2016), supports three biorepositories on the African continent, and facilitates policy and ethical recommendations (Consortium et al., 2014; Barchi and Little, 2016; Munung et al., 2016; de Vries and Pool, 2017).

Not only does Africa offer the richest genomic diversity in the world, it also has an extensive diversity of under-researched environmental exposures, including some exposures unique to the continent, which present important public health issues. Integration of genomic variants with environmental risk factors is vital to properly characterize disease risk in Africa. However, the starting point for incorporating genomic (G) and environmental (E) factors can be daunting. Important questions include: What environmental exposures are relevant to what African populations? What are the priorities? What has been studied and what are the relevant health outcomes? How do the exposures and health outcomes differ compared to populations in other regions of the world? How can genomics and environmental exposures be integrated?

The purpose of this review is to summarize and provide examples of the latest environmental health research and the G x E interactions that have been characterized this decade in Africa. In this paper we use the “G x E” terminology to broadly represent the integration of genomic and environmental data in a research project or study population. It can represent various statistical or data science methods for evaluating both genomic and environmental factors and is not strictly referring to the biological or statistical sense of the term interaction. Our review expands previous reviews describing the distribution of environmental exposures in selected African populations by focusing on the evaluated health outcomes related to environmental exposures and including all of Africa.

### Literature Search Strategy

We queried the PubMed database to identify peer-reviewed research or review articles or books (referred to generally as publications) examining environmental exposures and health outcomes in human populations residing on the African continent. We searched for publications evaluating the following environmental exposure categories: Ambient air pollution, indoor air pollution, electronic waste, environmental phenols, flame retardants, dietary mold, indoor mold, pesticides, perfluoroalkyl substances (PFASs), phthalates, and heavy metals. All search strategies, which included keywords as well as Medical Subject Headings (MeSH), are provided in the Supplementary Text, pulling preliminary results. All African countries were represented in the query and no exclusions were made based on the language of publication. The date range searched was from January 1, 2010 to March 20, 2018. Research articles were excluded if they did not include a measure/data for the queried exposure(s) and/or any health outcome(s). For example, research articles describing biomonitoring efforts or surveillance of human exposure to chemicals were not included if they did not also measure at least one health endpoint in a study population. We further refined our search to examine a subset of research or review articles that incorporated genomics, representing G x E research articles.

## Results

**Figure 1:**
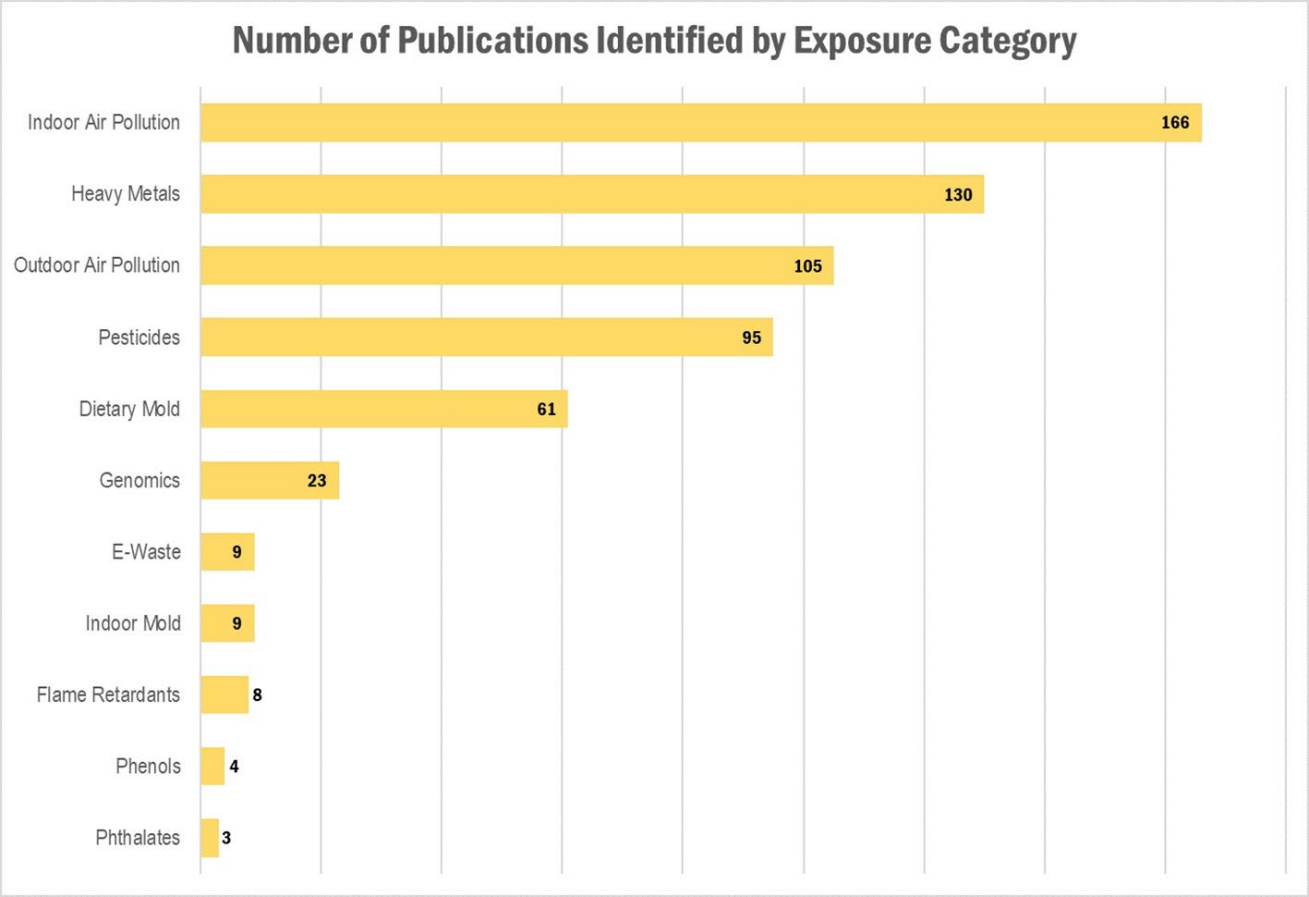
Results of the literature search: Number of publications identified, by exposure category. Publications could belong to more than one category.

**Figure 2:**
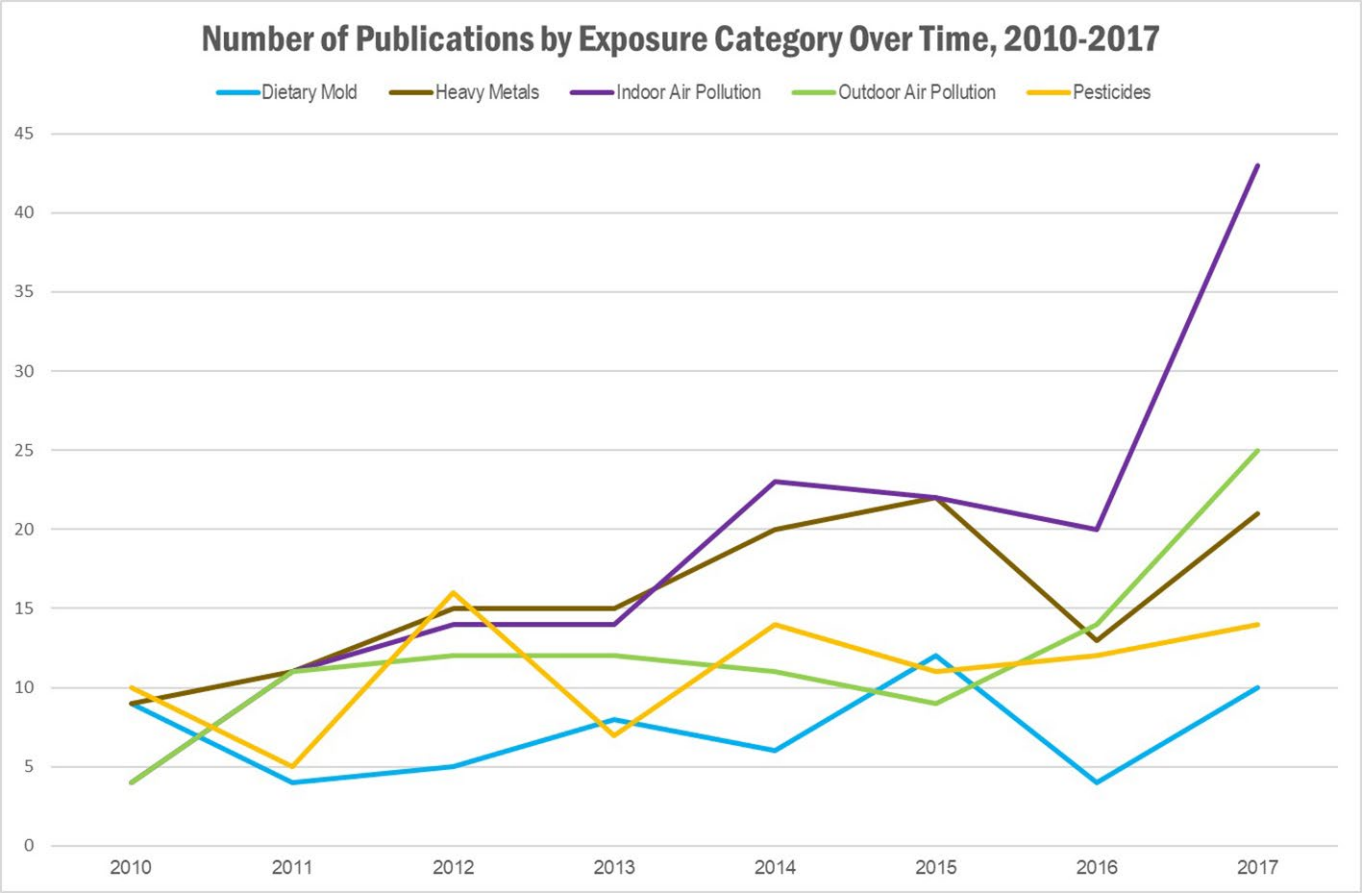
Results of literature search: Number of publications identified, by exposure category and year of publication. Publications could belong to more than one category.

Our literature search identified a total of 540 publications, representing 482 research articles, 57 reviews, and 1 book. A full list of the publications is provided in **Supplementary Table 1**. The results per exposure category are displayed in **Table 1** and **Figures 1** and **2**, where publications could belong to more than one category. The largest number of publications identified in our search represented exposures to indoor air pollution (n = 166), heavy metals (n = 130), ambient air pollution (n = 105), pesticides (n = 95), and dietary mold (n = 61). Notably fewer publications were retrieved for the exposure categories perfluoroalkyl substances (n = 16 initially, 0 after restricted to only those evaluating health outcomes), electronic waste (n = 9), indoor mold (n = 9), flame retardants (n = 8), environmental phenols (n = 4), and phthalates (n = 3). When we further subset the overall results to publications also evaluating genomic susceptibility or G x E interactions, we identified only 23 publications (21 research articles, 2 reviews, and no books). To summarize the publications across exposure categories, we highlight the important health endpoints, diseases, or outcomes evaluated, some specific exposures measured (and when possible, how measured), important at risk or vulnerable populations, and current research/data gaps.

**Table 1:** Summary of literature search results: Landscape of environmental health research in African populations. †

Exposure category	Example exposure sub categories ‡	Example sources of exposures	# Environmental health publications
Indoor air pollution	Particulate matter (PM2.5, PM10), carbon monoxide (CO), volatile organic compounds (VOCs), aeroallergens, dust mites, sulfur dioxide (SO2), nitrogen dioxide (NO2), black carbon (BC), polycyclic aromatic hydrocarbons (PAHs)	Cooking practices, cook stove type, environmental tobacco smoke, home heating practices, pests, domesticated and agricultural animals	166
Ambient air pollution	PM2.5, PM10, CO, SO2, NO2, ozone (O_3_), BC, PAHs	Vehicle emissions, wild fires, prescribed burning, wild fires, biomass burning, tobacco smoking, cooking, and factory emissions	105
Heavy Metals	Antimony, Arsenic, Cadmium, Chromium, Cobalt, Copper, Lead, Manganese, Mercury, Nickel, Selenium, Tin, Tungsten, Uranium, Zinc	Contaminated water, mining/occupational, diet, paint	130
Pesticides	Pyrethroids, organophosphates, organochlorines	Application of pesticides and exposure through agricultural occupations, indoor residual spraying, pest control	95
Dietary Mold	*A. flavus* and *A. parasiticus* producing aflatoxin in; Mycotoxins; cassava	Storage of staple foods such as groundnuts/peanuts, corn,	61
Indoor Mold	Airborne *Aspergillus* species (*A. niger*, and *A. flavus*, *A. fumigatus*)	Moist home/work conditions, flour mill and bakeries with grinding of grains	9
Electronic waste	Discarded electronic devices that can contain lead, cadmium, brominated flame retardants (BFRs), americium, mercury, hexavalent chromium, sulphur, perfluoroctanoic acid (PFOA), beryllium oxide	Discarded computers and accessories, mobile phones, audiovisual materials, or appliances	9
Environmental phenols	2,5-Dichlorophenol, Benzophenone-3 (Oxybenzone), Bisphenol A, Bisphenol F, Bisphenol S, Triclosan, Ethyl paraben, Propyl paraben, Butyl paraben	Plastics, food packaging, personal-care products	4
Flame retardants	PBDEs, brominated flame retardants (BFRs), TBBPA, hexabromocyclododecanes (HBCDs), OPFRs	Indoor furniture, recycled materials (e-waste related plastic casings)	8
Phthalates	Mono-benzyl phthalate, Mono-n-butyl phthalate, Mono-isobutyl phthalate, Mono-ethyl phthalate, Mono-(2-ethylhexyl) phthalate, Mono-(2-ethyl-5-hydroxyhexyl) phthalate, Mono-(2-ethyl-5-oxohexyl) phthalate, Mono-(2-ethyl-5-carboxypentyl) phthalate, Mono-(3-carboxypropyl) phthalate	Vinyl flooring, detergents, plastics, personal-care products, food packaging	3
Perfluoroalkyl substances (PFASs)	Perfluorooctane sulfonate, Perfluorooctanoic acid	Manufacturing, industry, exposure through fish consumption,	0

^†^Table sorted by number of publications and exposure category. Exposure category and example chemicals largely reflected the chemicals listed in the Fourth National Report on Human Exposure to Environmental Chemicals (https://www.cdc.gov/exposurereport/index.html). Exposures in the following categories were not included: Food safety, sanitation, waste management, personal or second-hand tobacco smoke, climate, or weather-related events.

^‡^Full details of subcategories can be found in **Supplementary Table 1** which details the search strategy.

## Indoor Air Pollution

We identified a total of 166 publications describing indoor air pollution and health endpoints across the African continent (**Table 1**). A 2016 *Lancet* review of 79 metabolic risk factors in a systematic analysis of the global burden of disease indicated that between 1990 and 2015, global exposure to household air pollution as well as unsafe sanitation, childhood underweight status, childhood stunting, and smoking, each decreased by more than 25% (GBD 2015 Risk Factors Collaborators, 2016). Household air pollution was listed as one of the top ten largest contributors to global disability-adjusted life-years (DALYs), representing 85.6 million (66.7 million to 106.1 million) global DALYs (2016).

### Health Outcomes

Across the indoor air pollution articles identified in our literature review, a critical health outcome noted was cardiovascular disease. Cardiovascular morbidities related to household air pollution have been identified in other countries, such as in China, Bangladesh, and Pakistan, raising ongoing concern for these risks in Africa (Noubiap et al., 2015). Studies specific to African populations identified in our review evaluated the impact of indoor air pollution on cardiovascular endpoints, such as cardiac chamber structure and function (Agarwal et al., 2018), blood pressure (Quinn et al., 2016; Alexander et al., 2017; Quinn et al., 2017; Arku et al., 2018; Swart et al., 2018), and inflammatory biomarkers (Olopade et al., 2017). Five of these articles focused on exposures to cooking or biomass fuel use in the home (Quinn et al., 2016; Alexander et al., 2017; Olopade et al., 2017; Quinn et al., 2017; Arku et al., 2018). Respiratory disease represented another major health outcome impacted by indoor air pollution; evaluated as the primary outcome of interest or a relevant co-morbidity in 77 of the identified indoor air pollution articles. This included articles describing general child respiratory health (Albers et al., 2015), acute lower respiratory tract infections in children (Buchner and Rehfuess, 2015), shortness of breath (Das et al., 2017), and asthma. Asthma and related morbidities were characterized in 37 articles and included outcomes such as asthma diagnosis and severity (Oluwole et al., 2017), asthma control (Kuti et al., 2017), allergen sensitization (Mbatchou Ngahane et al., 2016), and atopy (Morcos et al., 2011). Indoor air pollution-related impairments on innate immunity were also noted in some studies. For example, Rylance et al. (2015) observed an association between household air pollution and inflammatory responses assessed with IL6 and IL8 production and altered phagocytosis in macrophages exposed *in vitro* to respirable sized particulates.

### Exposures Measured

Most of the studies evaluating indoor air pollution focused on cooking practices including biomass fuel burning in indoor stoves. A total of 24 of the indoor air pollution research articles described exposure to dust. For example, dust was noted as a trigger for allergic rhinitis (Adegbiji et al., 2018) and house dust/dust mite exposure was associated with asthma (Bardei et al., 2016; Flatin et al., 2018). Particulate matter was evaluated in 24 of the indoor air pollution research studies, most focusing on PM10 (Abou-Khadra, 2013; Ibhafidon et al., 2014; Makamure et al., 2016; Jafta et al., 2017; Nkhama et al., 2017; Nkosi et al., 2017; Mentz et al., 2018) and PM2.5 exposures (Oluwole et al., 2013; Chafe et al., 2014; Ibhafidon et al., 2014; Dutta et al., 2017; Lacey et al., 2017; Lin et al., 2017a; Malley et al., 2017; Nkhama et al., 2017; Wylie et al., 2017a; Wylie et al., 2017b; Mentz et al., 2018). Some studies also measured NO, NO2, SO2, CO, and O3 (Jafta et al., 2017; Wylie et al., 2017a). DDT and DDE contamination from indoor residual spraying was found in household undisturbed dust and associated with DDT and DDE metabolites in serum of residents (Gaspar et al., 2015).

### At Risk Populations

Women conducting most of the household cooking and children helping or in proximity of cooking may be most impacted by indoor air pollution, depending on the family household practices.

### Research/Data Gaps

Although Rylance et al. (2015) described impairments to the immune system with exposure to indoor air, the interaction between this impairment and susceptibility to infections such as HIV or other infections warrants further research. A review by El-Gamal et al. (2017) describes literature on a wide range of aeroallergens across Africa but data on indoor aeroallergens are not included in all regions. The authors note the importance of characterizing genetic susceptibility in the context of immunodeficiencies in Africa, which has not received sufficient research attention.

## Ambient Air Pollution

### Health Outcomes

We identified 105 articles describing health impacts of ambient air pollution in Africa (**Table 1**). Nine of these represented review papers, covering outcomes such as chronic lung diseases among HIV positive individuals (Attia et al., 2017), children’s health such as pediatric asthma (Wolff et al., 2012; Jassal, 2015), biomarkers of genotoxicity (DeMarini, 2013), reproductive outcomes like preterm birth (Kumar et al., 2017; Malley et al., 2017), and severity of sickle cell disease (Tewari et al., 2015). Articles represented scientific depth and detail across the continent, covering key public health issues. Among all article types, notable endpoints evaluated were cardiovascular and cardiometabolic outcomes (Wichmann and Voyi, 2012; Benaissa et al., 2016), as well as broader burden of disease or life expectancy estimates (Berhane et al., 2016; Mokdad and GBD 2015 Eastern Mediterranean Region Lower Respiratory Infections Collaborators, 2018; Etchie et al., 2018). Some studies reported null findings. For example, an incremental life-time cancer risk was considered low in the context of exposure to PAHs from air pollution among city center residents of Kumasi, Ghana (Bortey-Sam et al., 2015). Additional outcomes evaluated included markers of oxidative stress, inflammatory cytokines, and chemokines (Cachon et al., 2014), chronic bronchitis from occupational exposures to dust (Hinson et al., 2016), elevated prostate specific antigen (PSA) among young men exposed occupationally to quarry pollutants (Ewenighi et al., 2017), chronic respiratory symptoms among limestone factory workers in Zambia (Bwalya et al., 2011), and allergic rhinitis in urban areas (Flatin et al., 2018). Exacerbation of silicosis due to higher doses of particulate matter exposure, impacts of exposure to prenatal air pollution on DNA methylation in the context of HIV status and antiretroviral treatment (Goodrich et al., 2016), asthma and asthma exacerbations, mortality, cerebrovascular outcomes, cardiovascular outcomes, and daily respiratory mortality were also evaluated.

### Exposures Measured

Several studies evaluated both indoor and ambient air pollution exposures and articles covered both the urban and rural settings (**Supplementary Table 1**). Ambient air pollution exposure in urban areas was noted in 36 publications including a study of air pollution and sleep disorders in children living in Egypt (Abou-Khadra, 2013). Occupational exposures were another important source of ambient air pollution exposure. Activities included limestone processing in Zambia (Bwalya et al., 2011), exposure to desert dust in West Africa [reviewed by de Longueville et al. (2013)], traffic exhaust (DeMarini, 2013), dust and fumes in artisanal mining (Ekosse, 2011), city transit-related air pollution (Elenge et al., 2011; Elenge and De Brouwer, 2011; Ekpenyong et al., 2012), stone quarrying industry exposures including deposition of inhaled aerosol particles at an industrial site in Egypt (Furi et al., 2017), sulfur dioxide (SO2) emissions from platinum group metal (PGM) smelting in Zimbabwe (Gwimbi, 2017), and charcoal processing activities in Namibia, including exposure to charcoal dust (Hamatui et al., 2016). DNA adducts to measure air pollution exposure among urban and suburban residents was also implemented in some studies (Ayi-Fanou et al., 2011).

### At Risk Populations

Across the articles evaluating air pollution exposure, occupationally exposed workers represented a critical population at risk. For example, exposure to pollutants through dust was mentioned in approximately one third of the ambient air pollution studies, half of which evaluated occupational exposures. Another study identified higher DNA adducts related to air pollution among taxi-motorbike drivers, roadside residents, street vendors, and gasoline sellers, compared to suburban and village inhabitants in Benin (Ayi-Fanou et al., 2011). Importantly, the impact of pollutant exposures correlating to occupation are not limited to impacts among workers. People living near work sites may also be affected. For example, Durban, South Africa represents one of Africa's busiest ports and the combination of industry, traffic, and biomass burning has led to substantial air pollution. A study of school children in Durban observed associations between air pollution exposures and respiratory symptoms, with notable burden on children with asthma (Mentz et al., 2018). These studies suggest that the impacts of occupational air pollution exposures are not limited to health endpoints in the workers alone. Immunocompromised individuals such as those living with HIV may also be more likely to experience chronic respiratory symptoms, abnormal spirometry, and chest radiographic abnormalities following air pollution exposures (Attia et al., 2017).

### Research/Data Gaps

Ambient air pollution exposure has been well characterized as an issue across Africa and around the world. Health impacts comparable to what has been identified in other populations were particularly clear for respiratory outcomes. Given the unique occupational settings in some regions of Africa, very high levels of exposure are of ongoing concern as is the peripheral impact on children and immunocompromised individuals.

## Heavy Metals

### Health Outcomes

Reproductive outcomes have been associated with various high heavy metal exposures in Africa. For example, associations between impaired semen quality and possible infertility has been reported for higher levels of cadmium, lead, zinc, and selenium (Awadalla et al., 2011; Oluboyo et al., 2012; Abarikwu, 2013; Famurewa and Ugwuja, 2017). Elevated serum heavy metals (cadmium and lead) along with a reduction of essential micronutrients (zinc and copper) may also contribute to recurrent pregnancy loss (Ajayi et al., 2012). An association between lower maternal zinc, copper, and cadmium levels as well as cord copper levels with low birthweight newborns has also been observed (Abass et al., 2014; Rollin et al., 2015). Elevated lead and arsenic exposures may be associated with preterm birth and other birth outcomes in general (Kumar et al., 2017; Rollin et al., 2017) and cord blood mercury was significantly associated with birth weight, length, and head and chest circumference in a Nigerian study population (Obi et al., 2015). Several African countries have a high level of preeclampsia and significant associations between preeclampsia and serum levels of calcium and magnesium or excretion of high amounts of several toxic metals, especially lead, have been identified (Ikechukwu et al., 2012; Motawei et al., 2013; Elongi Moyene et al., 2016). Egypt has one of the highest incidences of intrauterine growth retardation, and this appears to be positively correlated with heavy metal toxicity (El-Baz et al., 2015).

Lead toxicity (sometimes in combination with high cadmium exposures) has been shown to be associated with renal function impairment (Alasia et al., 2010b). Occupationally lead-exposed subjects have been shown to have significantly higher blood lead levels, as well as serum urea, creatinine, and serum uric acid levels, and other renal biomarkers and markers of nephrotoxicity. Multiple studies suggest a higher risk for developing hyperuricemia and renal impairment with high lead exposure (Alasia et al., 2010a; Cabral et al., 2012; Cabral et al., 2015). Workers in a variety of occupations, including automobile technicians, e-waste workers, miners, and shooting-range workers are at risk for substantially high lead levels (Saliu et al., 2015; Obiri et al., 2016b; Mathee et al., 2017). Blood lead levels in school children have been associated with a variety of behavioral and cognitive outcomes, including: lower IQ, poorer school performance, anti-social or violent tendencies, hearing deficiencies, and delayed onset of puberty (Naicker et al., 2010; Tomoum et al., 2010; Abdel Rasoul et al., 2012; Naicker et al., 2012; Kashala-Abotnes et al., 2016; AbuShady et al., 2017; Nkomo et al., 2017).

A high prevalence of acute lead poisoning in children has been an ongoing issue in many African countries (Bouftini et al., 2015; Bose-O’Reilly et al., 2018), with the lead poisoning crisis in Zamfara State, Northern Nigeria noted as one of the worst such cases in modern history. More than 400 children have died in Zamfara as a result of ongoing lead intoxication since early in 2010, and this acute lead poisoning is believed to be related to artisanal gold mining (Moszynski, 2010; Dooyema et al., 2012; Bartrem et al., 2014). Younger children with high venous blood lead level thresholds during the first year of the Zamfara outbreak response displayed a variety of neurological outcomes and were at higher risk for encephalopathy (Greig et al., 2014). Another recent lead poisoning outbreak reportedly occurred from consumption of an ayurvedic medicine in South Africa (Mathee et al., 2015).

A variety of cancers have also been associated with heavy metal exposure (Fasinu and Orisakwe, 2013; Obiri et al., 2016a; Obiri et al., 2016b). Low levels of selenium was associated with the development of breast cancer (Alatise et al., 2013), as was higher levels of lead for infiltrating ductal breast carcinoma (Alatise and Schrauzer, 2010). Cadmium and arsenic were found to be synergistically associated with bladder cancer and both exposures are often also associated with smoking status (Feki-Tounsi et al., 2013a; Feki-Tounsi et al., 2013b; Feki-Tounsi et al., 2014). A higher serum selenium concentration and a deficiency of zinc and molybdenum was found to be associated with esophageal squamous dysplasia (Ray et al., 2012; Pritchett et al., 2017). A positive association between cadmium exposure and pediatric cancer may also be present (Sherief et al., 2015). High levels of some heavy metals (chromium, nickel, cadmium) were associated with head and neck cancer as well (Khlifi et al., 2013a; Khlifi et al., 2013b).

Many studies reported neurological outcomes associated with occupational exposure to mercury. Prominent symptoms among fluorescent lamp factory workers exposed to mercury included tremors, emotional lability, memory changes, neuromuscular changes, and performance deficits in tests of cognitive function (Al-Batanony et al., 2013). Neurological symptoms, memory disturbances, and anxiety and depression were found in dentists exposed to mercury. Bilateral and symmetric intentional tremor in both upper limbs were found in dentists exposed to particularly high levels of mercury (Chaari et al., 2015). Chronic mercury intoxication, with tremor, ataxia and other neurological symptoms, along with kidney dysfunction and immunotoxicity, have been identified in individuals with high body burdens of mercury living in or near artisanal small-scale mining communities. Exposed groups showed poorer results in different neuropsychological tests. Over half of amalgam burners (workers with highest mercury levels as a group) were found to have symptoms of mercury intoxication (Bose-O’Reilly et al., 2017), and a large proportion of small-scale gold miners have mercury exposures above occupational exposure limits (Tomicic et al., 2011; Gibb and O’Leary, 2014; Steckling et al., 2014; Mensah et al., 2016).

The early effects of methylmercury due to fish consumption and other possible sources of exposure have also been extensively studied. Some negative outcomes associated with growth and nervous system effects on fetuses and newborns, cognitive function, reproduction, and longer-lasting cardiovascular effects as adults have been observed (Karagas et al., 2012; Gonzalez-Estecha et al., 2014). However, other nutrients, particularly n-3 polyunsaturated fatty acids (PUFAs) in fish, may modify some of these health effects (Lynch et al., 2011; Gribble et al., 2015; Strain et al., 2015). For example, although an adverse association of educational measures with postnatal mercury exposure in males but not females was found in one study from the Seychelles Child Development Study (Davidson et al., 2010), a number of other studies from this cohort have found no significant associations between methyl mercury exposure (either through fish consumption or prenatal exposure to dental amalgams) and neurodevelopmental outcomes (Watson et al., 2011; Watson et al., 2012; Watson et al., 2013; van Wijngaarden et al., 2013; van Wijngaarden et al., 2017).

A limited number of other studies have assessed various heavy metals and trace elements in relation to health outcomes. Alterations of some essential trace metals may play a role in the development of diabetes mellitus and obesity in children and older adults (El Husseiny et al., 2011; Harani et al., 2012; Azab et al., 2014; Badran et al., 2016). Arsenic and lead appear to impact diabetes and cardiovascular outcomes but have been studied very little in the African context (Ettinger et al., 2014). Exposure to arsenic was significantly associated with increased odds of asthma and tachycardia in one report (Bortey-Sam et al., 2018). Neurocognitive and motor impairments observed in konzo, a motor neuron disease associated with cassava cyanogenic exposure in nutritionally challenged African children, may possibly be driven by the combined effects of cyanide toxicity and selenium deficiency (Bumoko et al., 2015). Selenium and a number of other trace elements may also influence goiter development and general thyroid metabolism (Kishosha et al., 2011; Maouche et al., 2015; El-Fadeli et al., 2016; Gashu et al., 2016). Liver function may be compromised in nickel-plating workers (El-Shafei, 2011). Chronic neuropathology appears to be associated with chronic manganese exposure in South African mine workers (Gonzalez-Cuyar et al., 2014). Some trace metals may also play a role in the development of anemia (Henriquez-Hernandez et al., 2017). Low serum zinc levels were associated with acute lower respiratory infections (Ibraheem et al., 2014). Elevated blood lead levels seem to be associated with increased asthma severity (Mohammed et al., 2015). Selenium deficiency may be a risk factor for peripartum cardiomyopathy as well as other vascular complications and the impact of this may vary based on race (Karaye et al., 2015; Swart et al., 2018). An association of some metals with the risk of nasosinusal polyposis disease were observed for some genetic variants involved in DNA repair pathways affecting susceptibility (Khlifi et al., 2015; Khlifi et al., 2017). High concentrations of some harmful elements in geophagic clays eaten in Africa may be associated with cardiovascular outcomes (Olatunji et al., 2014). Mineral imbalances and lead exposure may also be associated with elevated blood pressure (Rebacz-Maron et al., 2013; Were et al., 2014). Disturbances in copper have been implicated in one study of Parkinson’s disease as well (Younes-Mhenni et al., 2013).

A connection between autism and various metals has also been studied. Altered urinary porphyrins, biomarkers of mercury toxicity, were observed in Egyptian children with autism spectrum disorder (Khaled et al., 2016). Levels of mercury, lead, and aluminum in hair of autistic patients was significantly higher than controls in one study (Mohamed Fel et al., 2015). High exposures of some heavy metals, particularly lead and mercury, have been treated with chelating agents, which appeared to improve autistic symptoms (Yassa, 2014).

### Exposures Measured

Mercury was sometimes determined by using a direct mercury analyzer, while most heavy metals were measured by atomic absorption spectrophotometer in blood and serum (and sometimes hair, nails, and air/soil samples) (Ojo et al., 2014; Were et al., 2014; Sherief et al., 2015; Iwegbue et al., 2017). The quantification of metals in various tissues was also assessed by atomic absorption spectroscopy (Feki-Tounsi et al., 2014). A variety of biomarkers were incorporated into various studies, especially to monitor kidney injury or dysfunction (Samir and Aref, 2011; Cabral et al., 2012; Cabral et al., 2015). Some heavy metals’ association with lipid peroxidation, DNA damage, oxidative stress, or apoptosis was assessed (El-Baz et al., 2015; Bortey-Sam et al., 2018) and the genotoxic impact of some occupational exposures was explored (El Shanawany et al., 2017).

### Vulnerable Populations

A variety of occupations clearly pose high risks for substantial exposure to heavy metals. Industrial metals are presently contaminating the environment and the water supplies, and the lack of education of workers and personal protective equipment was reported (Alatise and Schrauzer, 2010; Mensah et al., 2016). Individuals living near landfills and e-waste sites, particularly children, are at risk for a variety of exposures as e-waste components/constituents with heavy metal contamination can accumulate, in soil and surrounding vegetation, to toxic and genotoxic levels that could induce adverse health effects in exposed individuals (Alabi et al., 2012; Cabral et al., 2012). The outbreaks related to the fatal childhood lead poisoning illustrate the extreme vulnerability for young children (Dooyema et al., 2012; Bartrem et al., 2014). Other studies demonstrated the more subtle health outcomes related to lead exposures and suggest that even in the absence of overt clinical manifestations of lead toxicity, knowledge of lead exposure may influence the diagnosis in children presenting with anemia, intellectual impairment, poor academic performance, hearing impairments, and other outcomes (Abdel Rasoul et al., 2012).

### Research Gaps

There are numerous studies suggesting evidence for a variety of interactions among multiple heavy metals and trace elements, and the impact of these interactions on health outcomes. The interaction between lead and selenium is one of many interesting interactions associated with some cancers as lead may abolish the natural inhibitory effect on carcinogenesis observed for selenium (Alatise and Schrauzer, 2010). A synergistic interaction between cadmium and arsenic is also associated with bladder cancer (Alatise and Schrauzer, 2010; Feki-Tounsi et al., 2013a; Feki-Tounsi et al., 2014). There was evidence that obese children may be at a greater risk of developing an imbalance (mainly deficiency) of trace elements, which may be playing an important role in the pathogenesis of obesity and related metabolic risk factors (Azab et al., 2014). The mechanistic interactions of many heavy metals and trace elements, and the impact of these complex co-exposures for a variety of health outcomes is a substantial research gap in our current understanding.

The lead poisoning in Zamfara is an extreme example of both lead and multiple heavy metal mortality and morbidity, but highlights the importance of environmental remediation, chelation therapy, public health education, and control of mining activities to prevent future outbreaks (Dooyema et al., 2012; Bartrem et al., 2014). Furthermore, the primary source of lead pollution responsible for the lead poisoning of children in Nigeria appeared not to come from official mining activities but mainly from small scale operations conducted by local villagers, suggesting that some governmental regulation may be warranted (Moszynski, 2010). The oral chelating agent 2,3-dimercaptosuccinic acid (DMSA, succimer) appeared to be pharmacodynamically effective for the treatment of severe childhood lead poisoning in a resource-limited setting (Thurtle et al., 2014); in a number of situations, blood lead level monitoring has been used to show lower lead levels in children following implementation of such interventions (Brown et al., 2010; Bouftini et al., 2015).

The relationship between many metals and antioxidant enzymes and the role of the oxidative stress and inflammation pathways needs to be further explored (Maouche et al., 2015). Molecular mechanisms of how oxidative stress acts as a driver for organ dysfunction and the impact of antioxidants to mediate the potential toxic effect of various metal exposures will be important research areas to continue to explore (Samir and Aref, 2011). As one example, strategies to prevent konzo have successfully included dietary supplementation with trace elements, preferentially those with antioxidant and cyanide-scavenging properties (Bumoko et al., 2015).

The relationship between heavy metals and many disease outcomes are in preliminary stages in African studies and elsewhere. Other associations between heavy metals and some diseases have been established in predominantly European populations but have not been extensively studied in the African context. The association of metals with autism, respiratory disease, and other health outcomes have been inconsistent and will require additional exploration. The impact of other nutrients in fish modifying methylmercury neurotoxicity is also an ongoing source of investigation (Lynch et al., 2011).

## Pesticides

### Health Outcomes

Pesticides, particularly the insecticide DDT and its breakdown product dichlorodiphenyl trichloroethylene (DDE) and other endocrine disrupting compounds, have been associated with numerous reproductive outcomes including male infertility, impaired semen quality, increased sperm defects, anogenital distance, mean penile length in baby boys, various urogenital malformations, and spontaneous miscarriages and infant deaths (Lubick, 2010; Naidoo et al., 2010; El-Helaly et al., 2011; English et al., 2012; Abarikwu, 2013; El Kholy et al., 2013; Bornman et al., 2017). One recent paper suggested decreased ovarian reserve associated with exposure to pyrethroid pesticides (Whitworth et al., 2015). Emerging evidence suggests that many endocrine-disrupting pesticides have effects on cardiometabolic outcomes (Azandjeme et al., 2013). For example, DDT concentration has been consistently and positively associated with body composition and body weight in young girls, and DDT and DDE were found to be associated with elevated risk of hypertensive disorders in pregnancy (Coker et al., 2018; Murray et al., 2018), while chronic exposure of non-diabetic farmers to organophosphorus malathion pesticides appears to induce insulin resistance (Raafat et al., 2012). One study examined a variety of biochemical effects of pesticides including hematological profile, lipid parameters, serum markers of nephrotoxicity and hepatotoxicity, as well as the activities of butyryl cholinesterase (BChE), acetylcholinesterase (AChE), and thiolactonase-paroxonase (PON). The study concluded that long-term exposure to pesticides may play an important role in the development of vascular diseases *via* metabolic disorders of lipoproteins, lipid peroxidation and oxidative stress, inhibition of BChE, and decrease in thiolactonase-PON levels (Wafa et al., 2013).

Neurological outcomes were the most commonly associated health outcomes reported for cumulative exposure to both organophosphorus and pyrethroid compounds. Pesticide applicators and farm workers (including adolescent and child workers) exposed to these compounds exhibit neurological/neurobehavioral symptoms, deficits in neurobehavior performance tests, and neuromuscular disorders. These symptoms are often associated with greater inhibition of serum BChE and acetylcholinesterase activity, effect biomarkers often associated with neurotoxicity and cumulative TCPy, which is a biomarker of the organophosphorus pesticide chlorpyrifos (Sosan et al., 2010; Khan et al., 2014; Rohlman et al., 2014; Singleton et al., 2015; Manyilizu et al., 2016; Rohlman et al., 2016; Ismail et al., 2017b; Negatu et al., 2018). Some evidence for possible neurodevelopmental effects related to DDT in children has also been suggested (Osunkentan and Evans, 2015). Some associations were found between pesticide exposure and increased risks to various cancer outcomes, including bladder cancer, breast cancer, colorectal cancer, non-Hodgkin’s lymphoma, and hepatocellular carcinoma (Lo et al., 2010; Awadelkarim et al., 2012; Amr et al., 2015; Arrebola et al., 2015; VoPham et al., 2017). Respiratory outcomes were also commonly associated with both cumulative and acute pesticide exposure, including associations with idiopathic pulmonary fibrosis, decreased lung function/increased wheeze, lower airway inflammation, chronic cough, and asthma (Awadalla et al., 2012; Callahan et al., 2014; Ndlovu et al., 2014; Okonya and Kroschel, 2015; Mamane et al., 2016; Quansah et al., 2016; Sankoh et al., 2016). Interestingly, a novel Hirmi Valley liver disease was identified in recent decades in Ethiopa, which may be partially caused by co-exposure of acetyllycopssamine and DDT (Robinson et al., 2014). Perhaps most striking is the substantial literature on acute pesticide poisoning, both accidental and intentional, with adolescents’ intent on suicide (generally with the use of organophosphorus compounds and carbamates) contributing to an alarming increase in recent years (Balme et al., 2012; Azab et al., 2016; da Silva et al., 2016). In one study looking at acute pesticide poisoning in Kampala hospitals, 63% of cases of acute pesticide poisoning were intentional (Ssemugabo et al., 2017). The most common symptoms associated with accidental acute pesticide poisoning included skin and eye irritation, headaches, vomiting, nausea, chest pain respiratory disorders, and blurred vision (Karunamoorthi et al., 2012; Okonya and Kroschel, 2015; da Silva et al., 2016; Sankoh et al., 2016; Manyilizu et al., 2017; Ssemugabo et al., 2017).

### Exposures Measured

Many of the reviewed studies evaluated chronic pesticide exposure and alteration in serum enzymes associated with detoxification of pesticides, particularly inhibition of butyryl cholinesterase activity (Araoud et al., 2010; Araoud et al., 2011; Araoud et al., 2012). Biomarkers of exposures to the organophosphorus pesticides, chlorpyrifos (CPF) and Profenofos (PFF), were evaluated in some studies by measuring urinary levels of 3,5,6-trichloro-2-pyridinol (TCPy), a specific CPF metabolite and 4-bromo-2-chlorophenol (BCP), a specific PFF metabolite (Singleton et al., 2015). Inhibition of blood butyryl cholinesterase (BChE) and acetylcholinesterase (AChE) activities are effect biomarkers that were also evaluated in several of the reviewed studies (Ismail et al., 2010; Khan et al., 2014; Rohlman et al., 2014; Singleton et al., 2015; Rohlman et al., 2016; Ismail et al., 2017a; Ismail et al., 2017b). DDE/DDT was often assayed using ELISA (Bimenya et al., 2010).

### At Risk Populations

The *in utero* and early childhood effects of various pesticides and impact on long-term health highlights early life as a key susceptible time window for pesticide exposure. Adolescents working seasonally or during certain periods on farms may have a higher risk of neurotoxic effects of pesticide exposure because of their rapidly developing brains and bodies (Ismail et al., 2010; Ismail et al., 2017a; Ismail et al., 2017b). Because of the high morbidity and mortality associated with childhood and adolescent poisoning with pesticides (sometimes intentional), targeted prevention initiatives should be a high priority (Balme et al., 2010; Balme et al., 2012).

### Research/Data Gaps

The health effects of many pesticides have not been as extensively studied in African countries and may have different etiologies and patterns of exposure compared to other parts of the world. For example, the Sudan is experiencing a rapidly increasing cancer incidence, but little is known on tumor subtypes, epidemiology, or genetic or environmental cancer risk factors there or in other African countries (Awadelkarim et al., 2012).

Many of the reported agricultural pesticide studies in Africa were limited by exposure assessment methods (with many relying heavily on questionnaires alone to assess pesticide exposure and health risks). Future research could focus on improved pesticide exposure assessment methods, potentially incorporating multiple approaches and longitudinal studies to incorporate seasonal effects (VoPham et al., 2017). However, many opportunities exist now for comprehensive interventions to reduce both exposure and health risks associated with pesticide applications for both acute and cumulative exposures. 93% of farmers in rural Tanzania reported past lifetime pesticide poisoning (Lekei et al., 2014). Several reports have demonstrated acute pesticide poisoning to be associated with behaviors including lack of protective clothing, poor pesticide handling, not washing vegetables before eating, nozzle sucking, etc. (Magauzi et al., 2011; Oesterlund et al., 2014; Mekonen et al., 2015; da Silva et al., 2016; Sankoh et al., 2016; Manyilizu et al., 2017). One study from Sierra Leone reported most farmers having no knowledge about the safe handling of pesticides as 71% of them have never received any form of safety training (Sankoh et al., 2016). Comprehensive training and use of protective safety gear and clothing and safe handling practices may substantially reduce agricultural farmers’ health risks. In addition, given that chronic exposure to pesticides appears to affect several biochemical parameters, biomonitoring of effects in agricultural workers might be a useful way to assess the individual risk of handling pesticides. For example, BChE activity appears to be a useful indicator to monitor workers chronically exposed to pesticides as it is indicative of adverse effects of pesticides in agricultural workers and might detect the effects of pesticides before adverse clinical health effects occur (Araoud et al., 2011).

Important data are still needed to help policy makers perform risk-benefit analyses of the use of DDT and other pesticides in areas of Africa most heavily impacted by malaria (Thompson et al., 2018). A variety of indoor residual spraying of insecticides is associated with substantial decreased risk of developing malaria (Kigozi et al., 2012; Loha et al., 2012), and a recent study in South Africa reported DDT most effective for malaria control while acknowledging the detrimental health effects. Alternative prevention methods for controlling malaria are needed as well as more studies illustrating the long-term impacts of DDT on health (Hlongwana et al., 2013).

## Dietary Mold

### Health Outcomes

Mycotoxins, particularly aflatoxin and fumonisins, are natural toxins that many people in Africa are exposed to because they contaminate the staple diet of groundnuts, maize, and other cereals (Darwish et al., 2014). Aflatoxin in particular (which is produced by the fungi *Aspergillus flavus* and *Aspergillus parasiticus*) (Afum et al., 2016) is established as a cause of cirrhosis and human liver cancer (hepatocellular carcinoma-HCC) and growth faltering (perhaps due to micronutrient deficiencies) in young children (Obuseh et al., 2011; Bosetti et al., 2014; Shirima et al., 2015; Smith et al., 2015; Wirth et al., 2017). Adverse birth outcomes and anemia in pregnant women and acute aflatoxin poisoning in Africa are also concerns (Kimanya et al., 2010; Shuaib et al., 2010a; Shuaib et al., 2010b; Wild and Gong, 2010; Khlangwiset et al., 2011; Hoffmann et al., 2015). Several reports have investigated possible impaired semen quality (infertility) in men associated with aflatoxin (Abarikwu, 2013; Eze and Okonofua, 2015). There is potential association of zearalenone (a non-steroidal estrogenic mycotoxin) with breast cancer risk (Belhassen et al., 2015). Ergotism has been associated with several species of *Claviceps* that are in rye and other cereal grains (Belser-Ehrlich et al., 2013). Fumonisin B (1) is a mycotoxin produced by *Fusarium* spp. molds and it has been linked with primary liver cancer and esophageal cancer (Domijan, 2012). Fumonisins have also been associated with neural tube defects (Wild and Gong, 2010). Aflatoxin and other mycotoxins have been linked to possible neurotoxicological outcomes as well as chronic hepatomegaly (Gong et al., 2012). Ochratoxin A, a mycotoxin produced by several *Aspergillus* and *Penicillium* species, is associated with chronic interstitial nephropathy (Hmaissia Khlifa et al., 2012; Gil-Serna et al., 2018). Contaminated peanuts have been associated recently with growth faltering (Mupunga et al., 2017). Wheat handlers exposed to *A. flavus* may have elevated risks of liver cancer as well (Saad-Hussein et al., 2014). HIV positive and HBV/HCV positive individuals exposed to aflatoxin are at substantially increased disease risks due to the established synergistic action of aflatoxin with HIV and HBV/HCB infection (Kew, 2013).

### Exposures Measured

Aflatoxin has been established as a potent liver carcinogen working through a genotoxic mechanism involving metabolic activation to an epoxide, formation of DNA adducts and, in humans, modification of the p53 gene. Extensive mechanistic research combined with molecular epidemiology has allowed quantitative risk assessment for aflatoxin to be measured. Molecular biomarkers to quantify aflatoxin exposure in individuals were essential to link aflatoxin exposure with liver cancer risk. Biomarkers were validated in populations with high HCC incidence including the Gambia, West Africa region (Wogan et al., 2012). Aflatoxin metabolite AFM(1) and other mycotoxin metabolites have been measured in breast milk (Adejumo et al., 2013) while aflatoxin-albumin (AF-alb) and AFB1-lysine have typically been measured in blood plasma or serum through a variety of methods (See **Table 2**) (Mitchell et al., 2017; McMillan et al., 2018). Correlations between urinary aflatoxin M1 (AFM1) and aflatoxin albumin adduct (Af-alb) have been established and suggest that urinary AFM1 is a good biomarker of aflatoxin. AFM1 appears to measure shorter-term exposure to aflatoxin whereas AF-alb measures longer term exposure (Chen et al., 2018). Serum levels of ochratoxin A might also serve as a useful biomarker of HCC risk (Matsuda et al., 2013).

**Table 2:** Environmental exposures and health outcomes evaluated in African populations.

Exposure Category	Exposure Sub Categories	Exposure measurement methods	Health outcomes	Representative References
Indoor air pollution	Particulate matter (PM2.5, PM10)	Gent stacked filter unit sampler for collection of atmospheric aerosol in two size fractions (PM2.5 and PM10)	Pulmonary function and respiratory symptoms (couch, wheeze, shortness of breath, tightness in chest)	(Ibhafidon et al., 2014)
	Carbon monoxide (CO)	Draeger Carbon Monoxide 50/a-D (cumulative CO exposure 50–600 ppm-h) passive diffusion tubes (Draeger USA, Andover, MA) worn by study participants; measured length of color change in dosimeter tube after 72 hours and applied statistical models to determine cumulative and average exposure in ppm-h	Birthweight and related newborn anthropometrics (birth length, head circumference)	(Wylie et al., 2017a)
	Volatile organic compounds (VOCs)	Radiello passive samplers; passive thermal desorption VOC sampling tubes	Lifetime cancer risk; child health	(Moolla et al., 2015; Vanker et al., 2015)
	Aeroallergens	Skin prick tests to determine sensitization to aeroallergens	Allergic rhinitis, asthma symptoms, atopy	(Mbatchou Ngahane et al., 2016)
	SO2, NO2	Radiello passive samplers	Tuberculosis	(Jafta et al., 2017)
	Black carbon (BC)	Percentage of BC in alveolar macrophages, from bronchoalveolar lavage fluid (Rylance et al., 2015)	Lung microbiome	(Rylance et al., 2016)
	Polycyclic aromatic hydrocarbons (PAHs)	Urinary 1-hydroxypyrene (1-OHP), a biomarker for PAHs	Child neurocognition (delayed memory, attention scores, global cognition) in combination with HIV status	(Suter et al., 2018)
Ambient air pollution	Particulate matter (PM 2.5, PM10)	High volume cascade impaction air samples (Staplex 236, New York, USA described by Billet et al., 2007 (Billet et al., 2007)	Oxidative stress, inflammatory cytokines/chemokines, gene expression, secretion	(Cachon et al., 2014)
	CO	Exhaled CO with spirometry; portable carbon monoxide data logger	Respiratory symptoms; lung function	(Obaseki et al., 2014; Lawin et al., 2016; Lawin et al., 2017)
	SO2, NO2, O3, BC	AEROQUAL mobile air monitoring station to measure the ambient PM_10_ and SO_2_; SO2 emission trends related to smelting; NO2 and SO2 measured using a portable gas monitor	Self-reported coughing, nasal congestion, shortness of breath; related respiratory symptoms	(Gwimbi, 2017; Mbelambela et al., 2017; Morakinyo et al., 2017; Nkosi et al., 2017)
	PAHs	PAHs in street dust; atmospheric PAHs	Cancer risks	(Khairy and Lohmann, 2013; Obiri et al., 2013)
Heavy Metals	Zinc, copper, iron, calcium, selenium, chromium, lead, mercury, manganese, cadmium, cobalt, nickel, etc. (predominantly through serum but also hair, urine, semen, tissues, soil/dust)	Atomic absorption spectrophotometer methods, inductively coupled plasma mass spectrometry methods	Reproductive outcomes, renal dysfunction, neurological and neurobehavioral outcomes, cancers	(Feki-Tounsi et al., 2013a; Ojo et al., 2014; Sherief et al., 2015; Iwegbue et al., 2017)
	Lead, mercury, cadmium, chromium, zinc, iron, nickel, arsenic, manganese, etc. (soil and air samples)	X-ray fluorescence	Reproductive outcomes, renal dysfunction, neurological and neurobehavioral outcomes, cancers	(Bartrem et al., 2014; Ogundele et al., 2017)
	Lead, cadmium, chromium, copper, arsenic, tin, zinc, cobalt, etc. (soil samples)	Chemical analysis by the American Water Works Association	Reproductive outcomes, renal dysfunction, neurological and neurobehavioral outcomes, cancers	(Obiri et al., 2016a)
	Urinary porphyrins (biomarker of mercury exposure)	High-performance liquid chromatography	autism	(Khaled et al., 2016)
	Mercury	Direct mercury analyzer	Neurobehavioral outcomes	(Ojo et al., 2014)
	Selenium	Instrumental neutron activation analysis	Cancer	(Pritchett et al., 2017)
Pesticides	Acute pesticide poisoning	Plasma cholinesterase activity in blood can be measured using spectrophotometry to establish levels of poisoning by organophosphate and/or carbamates	skin and eye irritation, headaches, vomiting, nausea, chest pain respiratory disorders, and blurred vision; suicide	(Karunamoorthi et al., 2011; Magauzi et al., 2011; Okonya and Kroschel, 2015; da Silva et al., 2016; Ssemugabo et al., 2017)
	General agricultural	Hematological (hematocrits etc.), biochemical, and enzyme (creatine kinase, butyrylcholinersterase-BCHE, AChE, etc.) levels associated with detoxification of pesticides measured in serum; urinary levels of TCPy and BCP)	Neurological outcomes	(Araoud et al., 2010; Ismail et al., 2010; Araoud et al., 2011; Araoud et al., 2012; Rohlman et al., 2012; Khan et al., 2014; Singleton et al., 2014; Singleton et al., 2015; Ismail et al., 2017a)
	Fish and other contaminated wildlife	HCB, DDT, DDE, and PCBs measured with capillary gas chromatography with electron capture detector (persistent organochlorines in adipose tissues	Metabolic outcomes, cancer	(Achour et al., 2017; Thompson et al., 2017)
Dietary Mold	Aflatoxin metabolite (AFM(1))	In urine enzyme-linked immunosorbent assay (ELISA) method	Liver cancer, growth faltering	(Afum et al., 2016)
	Aflatoxin-albumin adduct (AF-alb)	enzyme-linked immunosorbent assay (ELISA) method ELISA or isotope dilution mass spectrometry (IDMS)	Liver cancer, growth faltering	(Castelino et al., 2015; McMillan et al., 2018)
	AFB1-lysine (AFB-Lys) adduct	determined *via* HPLC fluorescence in serum or high resolution LC-MS/MS with IDMS	Liver cancer, growth faltering	(Kang et al., 2015; McMillan et al., 2018)
	Various mycotoxins and fungal metabolites [including aflatoxins (AFB1, AFB2, AFG1, AFG2 and AFM1), beauvericin (BEAU), cyclopiazonic acid (CPA), moniliformin, nivalenol and ochratoxin A]	high-performance liquid chromatography in plasma or liquid chromatography tandem mass spectrometry in groundnuts	Liver cancer, growth faltering	(Matsuda et al., 2013; Oyedele et al., 2017)
	Fumonisin B1 (UFB1)	liquid chromatography-mass spectrometry in urine	Liver cancer, growth faltering	(Shirima et al., 2015)
Indoor Mold	Aspergillus (airborne)	Isolated from dust in work settings (bakeries, mills)	AFB1, serum albumin, liver enzymes (aspartate aminotransferase, alanine aminotransferase, alkaline phosphatase)	(Saad-Hussein et al., 2016)
	Fungal spores (*A. niger*, and *A. flavus*, *A. fumigatus*, etc.)	MALDI-TOF mass spectrometry from air samples	Liver cancer, growth faltering	(Niare-Doumbo et al., 2014)
Electronic waste	PAHs, PCBs, PBDEs	Gas chromatography/spectrophotometry	Cancer risks, hearing difficulties, cardiovascular disease symptoms	(Alabi et al., 2012)
	DNA damage	In peripheral blood lymphocytes using alkaline comet assay	Cancer risks, hearing difficulties, cardiovascular disease symptoms	(Alabi et al., 2012)
	Lead, cadmium, chromium, copper, arsenic, zinc, etc.	Chemical analysis prescribed by the American Water Works Association	Cancer risks, hearing difficulties, cardiovascular disease symptoms	(Obiri et al., 2016a)
Environmental phenols	PBDEs	Measured in breast milk	Birthweight and birth length (increased)	(Muller et al., 2016)
	PBDEs, PCBs, OCPs	Chemicals or chemical metabolites measured in serum and blood samples	No statistically significant associations between contaminants and colorectal cancer	(Motsoeneng and Dalvie, 2015; Muller et al., 2016; Abou-Elwafa Abdallah et al., 2017; Kumar et al., 2017)
Flame retardants	PBDEs, HBCD, 1,2-bis(2,4,6-tribromophenoxy) ethane (BTBPE), hexabromobenzene (HBB), (2,3-dibromopropyl) (2,4,6-tribromophenyl) ether (DPTE), pentabromoethylbenzene (PBEB) and 2,3,4,5,6-pentabromotoluene (PBT)	Measured in breast milk and self-reported Pemba during pregnancy	Birth outcomes	(Muller et al., 2016)
	Organohalogenated contaminants (OCs) including organochlorine pesticides (OCPs), polychlorinated biphenyls (PCBs) and PBDE)	Measured in soil; blood	Risk assessment for various adverse health outcomes; cancer risks	(Sun et al., 2016; Abou-Elwafa Abdallah et al., 2017)
	PBDEs	gas chromatography electron impact ionization mass spectrometry	Hazard quotients (general health risks)	(Akortia et al., 2017)
Phthalates	Monoethyl phthalate (MEP)	Urinary concentrations of phthalate metabolites; food storage questionnaire	Did not evaluate a health outcome†	(Colacino et al., 2011)
	Dibutyl phthalate (DBP), di(2-ethylhexyl) phthalate (DEHP), diisononylphthalate (DINP)	Measured in drinking water; measured in urine using enzymatic deconjugation of the metabolites from their glucuronidated form, solid-phase extraction, separation with high performance liquid chromatography, and detection by isotope-dilution tandem mass spectrometry as described previously (Silva et al., 2007)	Human health risk assessment	(Van Zijl et al., 2017)
Perfluoroalkyl substances (PFASs)	PFOS	Measured in maternal serum and cord blood; PFCs analyzed using a QTOFmicro quadropoletime-of-flight (QTOF) mass spectrometer, as described by Rylander et al. PFOS was quantified and reported as the sum of the unspecified branched PFOS and linear PFOS.(Rylander et al., 2009)	Did not evaluate a health outcome†	(Hanssen et al., 2010)

^†^ Included in the table as an example study measuring exposure in humans but not health outcomes. We did not retrieve any studies specific to PFAS in African populations measuring both exposures and health endpoints in humans.

### At Risk Populations

Growth faltering makes young children particularly vulnerable to mycotoxins as fetal and early postnatal growth and development appear to be affected and because aflatoxin is known to cross the placental barrier (Castelino et al., 2014; Castelino et al., 2015). Interventions should focus on reducing mold exposures during critical periods of fetal and infant development, particularly for nursing infants having possible contaminated milk (Adejumo et al., 2013). HIV positive and HBV/HCV positive individuals are also at risk populations for the health effects related to aflatoxin exposure (Kew, 2013). Agricultural workers and rural populations, particularly subsistence farming communities, are important at risk populations as well.

### Research/Data Gaps

Mycotoxin risk management has been successful in West Africa and other African countries, and this has substantially reduced disease attributable to aflatoxin (Liu et al., 2012; McGlynn et al., 2015). Many intervention/prevention efforts (including post-harvest storage measures) are now underway to reduce exposure to highly toxic and carcinogenic contaminants in staple diets in Africa, especially aflatoxin and fumonisins, which people are exposed to daily through grain and cereal staples in their diet. Aflatoxin biomarkers have also been used to show that primary prevention to reduce aflatoxin exposure can be achieved by low-technology approaches at the subsistence farm level in sub-Saharan Africa (Wogan et al., 2012). Daily urinary AFM1 levels have been shown to be useful as a biomarker of internal aflatoxin B1 exposure in short-term intervention trials to determine efficacy of interventions (Mitchell et al., 2013). Further application of knowledge to practice is currently underway with numerous intervention/prevention studies, clinical trials, and education (Wild and Gong, 2010; Hoffmann et al., 2015; Saleh et al., 2015). The comprehensive approach used to create many successful preventive interventions to reduce health risks associated with aflatoxin is a model for the development, validation, and application of biomarkers for other environmental exposures (Wogan et al., 2012).

There is evidence that maternal exposure to aflatoxin during the early stages of pregnancy is associated with differential DNA methylation patterns of infants, including in genes related to growth and immune function but how mycotoxin exposure in embryonic and fetal development may influence later disease risk needs to be explored (Hernandez-Vargas et al., 2015). The association between aflatoxin exposure and alteration in immune responses observed in humans suggest that aflatoxin could suppress the immune system and work synergistically with HIV to increase disease severity and progression to AIDS, but in general, the neurotoxicological and immunological/immunodepression aspects are not well understood (Jolly et al., 2015). While studies have shown synergism between aflatoxin and HBV in causing HCC, much less is known about whether aflatoxin and HCV synergize similarly (Palliyaguru and Wu, 2013). The relationship between HIV transmission frequency and fumonisin contamination also needs to be explored (Williams et al., 2010). Childhood immunizations for hepatitis B in many West African countries is still lagging behind many other countries, and this vaccination alone could substantially impact health risks (Ladep et al., 2014). Some findings of significant decrease in vitamin A associated with AF-ALB suggest that aflatoxin exposure compromises the micronutrient status of people who are immunocompromised, including people living with HIV (Obuseh et al., 2011). The interaction between aflatoxin and micronutrient deficiencies warrants more investigation (Watson et al., 2016; Watson et al., 2017).

## Indoor Mold

### Health Outcomes

Indoor fungal-related outbreaks were measured and found to be associated with mucormycosis, endophthalmitis, aspergillosis, as well as asthma exacerbation and other infections in a variety of Sub-Saharan African samples (Gharamah et al., 2012; El-Mahallawy et al., 2016).

### Exposures Measured

Indoor mold was primarily measured as fungal spores present in airborne samples and measured in nasal swabs and sputum samples (Niare-Doumbo et al., 2014; Diongue et al., 2015) (**Table 2**).

### At Risk Populations

At risk-populations that were examined included pediatric wards with leukemia patients and other immunocompromised or allergic patients, oncology wards, and ophthalmology operating rooms (Gharamah et al., 2012; Niare-Doumbo et al., 2014; Gheith et al., 2015). Occupational exposure to aflatoxin was found in textile workers and was associated with liver tumor biomarkers (Saad-Hussein et al., 2013). Airborne *Aspergillus* was associated with higher serum aflatoxin B1 and several liver enzymes among workers handling wheat flour (Saad-Hussein et al., 2016) as well, suggesting workers for several occupations may be at increased risk for indoor mold exposures.

### Research/Data Gaps

Different sensitization rates have been observed in different classes of patients. Highest indoor mold counts in many studies were often associated with the rainy season but more research exploring sensitization rates and seasonal variations is needed (Hasnain et al., 2012). Protective gear and safety measures to reduce exposure for some occupations are needed.

## PFAS

The literature describing PFAS-related health outcomes in Africans was extremely limited. Although our review did not find research articles evaluating PFAS and health outcomes in African populations, there has been increasing attention to PFAS exposure, including studies measuring PFAS in non-humans [e.g. crocodiles, fish (Ahrens et al., 2016)]. One study evaluated PFAS in maternal serum and cord blood in South Africa (Hanssen et al., 2010) but did not evaluate specific health endpoints in the study population where PFAS was measured.

### Health Outcomes

Ahrens et al. (2016) described a risk assessment strategy for evaluating potential human health outcomes related to the PFAS levels in different compartments of Ethiopia’s largest lake, Lake Tana. Their findings do not indicate any elevated health risks, but the authors note the potential for harmful effects with increasing levels over time.

### Exposures Measured

Across the reviewed studies, perfluoroalkyl acids (PFAAs) were measured in water, sediment, and fish in Lake Tana, Ethiopia (Ahrens et al., 2016), in tilapia in South Africa (Bangma et al., 2017), and in wastewater and sludge from selected wastewater treatment plants in Kenya (Chirikona et al., 2015). Another study measured PFCs in maternal serum and cord blood of South African women-infant pairs. They did not report specific health outcomes but did note that the median maternal PFOS concentration was lower than has been reported in other studies, whereas the PFOA concentration was the same. The authors suggested that different exposure pathways (and sources) exist in this population compared to western-style study populations (Hanssen et al., 2010).

### At Risk Populations

Individuals with high fish consumption (e.g. living near the lake, depending on the lake for food or occupation, etc.) are at higher risk of these exposures. Although the results in our review did not evaluate specific health outcomes, PFOS levels were reportedly increasing between 1978 and 2001 in a study population in Southern Sweden that included women from countries of origin within and outside of Sweden, including Africa. This study observed higher levels in women with Sweden as the country of origin, compared to women from the Middle East, North Africa, and sub-Saharan Africa (Ode et al., 2013). Ode et al. report that PFOS levels increased over time, whereas PFOA and PFNA levels were unchanged between 1978 and 2001 in their study population.

### Research/Data Gaps

More research incorporating exposures and health endpoints measured in the same study population in Africa are needed. This gap may reflect potentially lower levels in African populations compared to U.S. and European populations where PFAS health studies have focused. However, as industrialization, urbanization, and globalization contribute to growing ubiquity of many environmental chemical exposures, we anticipate PFAS exposures may increase in African populations.

## Electronic Waste

### Health Outcomes

A variety of crude recycling operations in developing nations, including Africa, have been reported to lead to multiple health risks. In many cases, e-waste workers are exposed to highly contaminated fumes due to burning practices (Akormedi et al., 2013). Self-reported hearing difficulties and stress associated with potential cardiovascular disease symptoms (including elevated blood pressure levels) have been reported in electronic waste recycling workers (Were et al., 2014; Burns et al., 2016). Workers burning e-waste products have been reported as having very high blood lead levels and noise exposures often exceed recommended occupational and community noise exposure limits (Burns et al., 2016). Workers have reported moderate to prominent levels of perceived stress as measured *via* Cohen’s Perceived Stress Scale (Burns et al., 2016). Higher levels of a few chemicals related to e-waste recycling have also been associated with increased cancer risks (Obiri et al., 2016a).

### Exposures Measured

Across the e-waste studies reviewed, levels of polyaromatic hydrocarbons (PAHs), polychlorinated biphenyls (PCBs), and polybrominated diphenyl ethers (PBDEs) were typically analyzed using gas chromatography/spectrophotometry. Heavy metals were measured using atomic absorption spectrophotometry, and DNA damage was assayed in human peripheral blood lymphocytes using an alkaline comet assay in soil and plant samples (Alabi et al., 2012). Lead, cadmium, chromium, copper, arsenic, tin, zinc, and cobalt *via* oral and dermal contact in bottom ash and soil were measured using random sampling techniques and analyzed using standard methods for chemical analysis prescribed by the American Water Works Association (Obiri et al., 2016a) (**Table 2**).

### At Risk Populations

In general, e-waste workers in many African countries are a vulnerable at-risk population that may have a limited social safety net or legal protections. The chemical exposures reported in e-waste studies are relevant not just to e-waste workers but also to traders and residents, including children living in neighboring areas.

### Research/Data Gaps

The exposures related to e-waste recycling is an understudied area but limited reported studies suggest clear health risks associated with this activity. Cleaner technologies and protective gear for workers as well as education efforts are needed. Several reports recognized the complicated e-waste infrastructure system in some African countries and the need to understand all stakeholders involved (Amankwaa et al., 2017). One review suggested approaching the e-waste crisis in sub-Saharan Africa with an ongoing health impact assessment that would address the health, environmental, and social aspects of the issue and where all the steps of the assessment are performed with input from local communities (Tetteh and Lengel, 2017).

## Flame Retardants

### Health Outcomes

Several recent African studies have quantified concentrations of a variety of flame retardants and attempted to associate exposure levels with different health outcomes. Elevated levels of concentrations of polybrominated diphenyl ethers (PBDEs), polychlorinated biphenyls (PCBs) and some organochlorine pesticides (OCPs) were not found in colorectal cancer patients in Egypt, compared to controls (Abou-Elwafa Abdallah et al., 2017). Potential health concerns related to estimated lifetime cancer risk and other risks were suggested for levels of some organochlorine pesticides observed in soil samples (Sun et al., 2016), as well as DDT and PCBs from dietary fish exposure in one study (Ben Ameur et al., 2013). However, other studies did not show levels of flame retardants exceeding safety guidelines from dietary fish intake (Asante et al., 2013; El Megdiche et al., 2017). Concerns related to levels of PCBs, as well as brominated flame retardants such as polybrominated diphenyl ethers (PBDEs) and hexabromocyclododecanes (HBCDs), hexabromobenzene (HBB), 2,3-dibromopropyl-2,4,6-tribromophenyl ether (DPTE), pentabromoethylbenzene (PBEB) and 2,3,4,5,6-pentabromotoluene (PBT), were also measured in breast milk in several studies and found to be unexpectedly high (with estimated hazard quotient values exceeding the threshold of 1 or the US EPA reference doses exceeded) (Asante et al., 2011; Muller et al., 2016).

### Exposures Measured

The concentrations of polybrominated diphenyl ethers (PBDEs) were commonly measured in the reviewed studies by using gas chromatography electron impact ionization mass spectrometry (Akortia et al., 2017).

### Vulnerable Populations

Potential health risks for children, particularly nursing infants, for a variety of flame retardants were observed. PCBs in dirty oils and obsolete equipment as well as new sources of DDT for malaria control in some countries in Africa were noted as potential sources of exposure (Asante et al., 2011; Sun et al., 2016).

## Phenols

Only four studies met the inclusion criteria for this review of measuring phenols in relation to health outcomes in Africa (Motsoeneng and Dalvie, 2015; Muller et al., 2016; Abou-Elwafa Abdallah et al., 2017; Kumar et al., 2017), one of which covered the topic in a recent review of environmental factors and global estimates of preterm birth (Kumar et al., 2017). Abou-Elwafa et al. (2017) measured polychlorinated biphenyls (PCBS), some organochlorine pesticides (OCPs), as well as polybrominated diphenyl ethers (PBDEs, see flame retardants section) in serum of study participants in Egypt. Notably, concentrations of these chemicals were much lower in this Egyptian study population compared to other published concentrations in populations around the world.

### Health Outcomes

The health outcomes evaluated included colorectal cancer (Abou-Elwafa Abdallah et al., 2017), preterm birth (Kumar et al., 2017), birth weight and birth length (Muller et al., 2016), and neurological endpoints such as difficulty with buttoning, reading, or writing notes (Motsoeneng and Dalvie, 2015).

### Exposures Measured

Across the studies, phenols were measured in serum, breast milk, and urine. Some of these studies also measured PCBs and OCPs and are discussed in greater detail in other sections.

### At Risk Populations

Similar to other chemical exposure categories, high risk populations include pregnant women, nursing infants (early life exposures in general), and young children.

### Research/Data Gaps

The limited publications describing phenols and health outcomes in Africa likely reflect the limited data of phenol use, distribution, and concentrations in human urine, serum, or blood. Despite the variability in the use of these compounds in some regions, the lipophilic and persistent characteristics of some chemicals enable bioaccumulation in the food chain. Most are listed as persistent organic pollutants under the United Nations Environment Programme (UNEP) Stockholm Convention (UNEP, 2009) (https://www.wipo.int/edocs/lexdocs/treaties/en/unep-pop/trt_unep_pop_2.pdf). There is very limited data for Africa evaluating health outcomes related to phenols. However, several studies document the existence of phenols in human samples such as methylated polybrominated diphenyl ethers in human milk from Bizerte, Tunisia (Ben Hassine et al., 2015), dust exposure in Egypt (Hassan and Shoeib, 2015), and urinary bisphenol A (not persistent) concentrations in girls in rural and urban Egypt (Nahar et al., 2012). The levels in Egypt were lower than NHANES age-matched American girls but the authors noted associations with food storage in plastic containers which may change over time in some Africa regions.

## Phthalates

### Health Outcomes

Data on the health effects of phthalates in Africa was also extremely limited—only three articles retrieved in our literature search evaluated the impact of exposure to phthalates and any health outcomes in an African study population (Colacino et al., 2011; Kumar et al., 2017; Van Zijl et al., 2017). Adverse health outcomes evaluated in these articles were preterm birth (Kumar et al., 2017) and estrogenic activity (Van Zijl et al., 2017). The third study focused on sources of exposure to phthalates among premenstrual girls in Egypt, reporting BMI, waist and hip circumference, and other anthropometric characteristics, comparing rural and urban study participants. The authors also compared the phthalate levels in this Egyptian population to the age-matched girls in U.S. NHANES data, identifying key sources of exposure (Colacino et al., 2011). Storage of food in plastic containers was a statistically significant predictor of mono-isobutyl phthalate (MiBP) measured in urine of premenstrual girls, suggesting an important dietary route of exposure. The urinary measurements of phthalates were similar between the US and Egyptian age-matched girls (Colacino et al., 2011). Kumar et al. (2017) reviewed potential contributing factors to preterm birth and suggested phthalates should be evaluated more extensively in Africa.

### Exposures Measured

Phthalates were measured in urine using enzymatic deconjugation of the metabolites from their glucuronidated form, solid-phase extraction, separation with high performance liquid chromatography, and detection by isotope-dilution tandem mass spectrometry as described previously (Silva et al., 2007; Van Zijl et al., 2017). Estrogenic activity was identified in drinking water from Pretoria and Cape Town that also contained detectable levels of estrogens, bisphenol-A, and phthalates. No harmful effects from these were detected in their study population—the health risk assessment revealed acceptable health and carcinogenic risks associated with the consumption of distribution point water.

### At Risk Populations

Early life exposure is an important consideration in this group, impacting pregnant women and young children.

### Research/Data Gaps

Much more work is needed to evaluate the health implications from exposure to phthalates in the African setting, as exposures may increase over time.

## G x E and Related Integration of Genomic and Environmental Exposures

Only 23 of the identified studies in our literature review considered both genomic and environmental factors related to health outcomes in Africa. All of these articles are listed in **Table 3**. Although effects of *PON1* genotype on organophosphorus pesticide chlorpyrifos (CPF) exposure effects for Egyptian agricultural workers were found to be minimal (Ellison et al., 2012), several other studies reported significant effects of genotype modification for various exposure risks. The *GSTP1* genotype appeared to modify the effects of ambient air pollutants PM10 and SO2 on lung function in South African children (Reddy et al., 2012). Genetic polymorphisms in *NAPH* and *SOD2* may modulate pesticide-associated risk for bladder cancer (Amr et al., 2015). The *TNF*-alpha 308 polymorphisms were associated with increased effects on lung function for several pollutants (SO2 and NO2) (Makamure et al., 2016). *PON1* 192RR and *CYP2D6* 1934A alleles were found to potentially alter susceptibility to organophosphate chronic toxicity in Egyptian agricultural workers as well (Tawfik Khattab et al., 2016). *ERCC3* and *ERCC2* polymorphisms impact the effect of cadmium exposure for nasal polyposis (Khlifi et al., 2017). Air pollution’s effect on cardiovascular risk factors may be modulated by the *APOA5* 1131 polymorphism (Lin et al., 2017b). The *CD14* CT/TT genotype appears to be protective for increased exposure to some ambient air pollutants (Makamure et al., 2017). DNA variants in *NAT2*, *PON1*, and *GSTM1* may also modify organophosphate neurotoxicity (Glass et al., 2018).

**Table 3:** G x E † and health outcomes evaluated in African populations.

Reference	Title	Exposure(s) Measured	Genomic factors or Biomarkers measured	Health outcomes
Ayi-Fanou et al. (2011)	DNA-adducts in subjects exposed to urban air pollution by benzene and polycyclic aromatic hydrocarbons (PAHs) in Cotonou, Benin	DNA adduct levels (benzene and PAHs)	Genetic susceptibility from adduct level variation	Cancer, chronic respiratory, etc.
Ellison et al. (2012)	*PON1* status does not influence cholinesterase activity in Egyptian agricultural workers exposed to chlorpyrifos	Pesticides (chlorpyrifos)	*PON1* genotypes	Cholinesterase activity
Reddy et al. (2012)	*GSTM1* and *GSTP1* gene variants and the effect of air pollutants on lung function measures in South African children	Ambient air pollutants (SO2), NO2, NO, and PM10)	*GSTM1* (glutathione-S-transferase M1 gene) and *GSTP1* (glutathione-S-transferase P1 gene)	Asthma
DeMarini (2013)	Genotoxicity biomarkers associated with exposure to traffic and near-road atmospheres: a review	Exposure assessments (protein adducts, 1-hydroxypyrene, or polycyclic aromatic hydrocarbons)	Genomic biomarkers (gene expression, leukocyte telomere length, DNA methylation), cytogenetic markers, DNA damage markers	Cancer
Kew (2013)	Aflatoxins as a cause of hepatocellular carcinoma	Aflatoxin	Arginine to serine mutation at codon 249 of the p53	Hepatocellular carcinoma
Castelino et al. (2014)	Seasonal and gestation stage associated differences in aflatoxin exposure in pregnant Gambian women	Aflatoxin	*R249S* mutation in p53, *CYP3A4* and *CYP3A5* isoforms	Hepatocellular carcinoma
Kabasenche (2014)	DDT, epigenetic harm, and transgenerational environmental justice	Dichlorodiphenyltrichloroethane (DDT)	Epigenetics	Developmental abnormalities, reproductive disease, neurological disease, and cancer
Myers et al. (2014)	Neurotoxicology and development: human, environmental and social impacts	Metals, solvents, pesticides	Genetic/epigenetic biomarkers	Neurodevelopmental outcomes
Amr et al. (2015)	Pesticides, gene polymorphisms, and bladder cancer among Egyptian agricultural workers	Pesticides	Genetic polymorphisms for *NAD(P)H*: quinone oxidoreductase I (*NQO1*) and superoxide dismutase 2 (*SOD2*)	Bladder cancer
Hernandez-Vargas et al. (2015)	Exposure to aflatoxin B1 in utero is associated with DNA methylation in white blood cells of infants in The Gambia	Aflatoxin	Genome-wide methylation/differential methylation of *FGF12*, *IGF1*, *CCL28*, *TLR2*, and *TGFB1*	Liver cancer, growth stunting
Goodrich et al. (2016)	Prenatal exposures and DNA methylation in newborns: a pilot study in Durban, South Africa	Air pollution and HIV	Differential methylation related to xenobiotic metabolism, cytochrome p450, chemical stimuli detection, and viral regulation pathways	Developmental outcomes
Makamure et al. (2016)	Tumor necrosis factor alpha polymorphism (*TNF-308alpha G/A*) in association with asthma related phenotypes and air pollutants among children in KwaZulu-Natal	Ambient air pollutants such as SO2, NO2, NO, and PM10	Tumor necrosis factor alpha polymorphism (*TNF-308alpha G/A*)	Lung function relevant to asthma and airway inflammation
Rylance et al. (2016)	Household air pollution and the lung microbiome of healthy adults in Malawi: a cross-sectional study	Black carbon particulates	Lung microbiome	Respiratory diseases/infections
Tawfik Khattab et al. (2016)	The role of *PON1* and *CYP2D6* genes in susceptibility to organophosphorus chronic intoxication in Egyptian patients	Pesticides (organophosphorus compounds)	*PON1 Q192R* and *CYP2D6 G1934A*	Neurological symptoms associated with chronic organophosphate toxicity
El Okda et al. (2017)	Immunological and genotoxic effects of occupational exposure to alpha-cypermethrin pesticide	Pesticides (alpha-cypermethrin)	P53 mutations and antioxidant measures of superoxide dismutase (*SOD*), catalase (*CAT*), glutathione (*GSH*) and glutathione peroxidase (GPx)	Immunological and genotoxic outcomes
El Shanawany et al. (2017)	The potential DNA toxic changes among workers exposed to antimony trioxide	Occupational exposure to antimony trioxide	DNA damage (apurinic/apyrimidinic sites)	Genotoxic impact
El-Gamal et al. (2017)	Allergy and immunology in Africa: Challenges and unmet needs	Airborne viruses, smoke, indoor dampness, cockroaches	aeroallergens	Allergic and immunodeficiency diseases
Khlifi et al. (2017)	Gene-environment interactions between *ERCC2*, *ERCC3*, *XRCC1* and cadmium exposure in nasal polyposis disease	Cadmium	*ERCC3*, *ERCC2*, and *XRCC1*	Nasal polyposis
Lin et al. (2017b)	*APOA5* Gene Polymorphisms and Cardiovascular Diseases: Metaprediction in Global Populations	Air pollution	Apolipoprotein A5 (*APOA5*) 1131	Cardiovascular disease
Makamure et al. (2017)	Interaction between ambient pollutant exposure, *CD14* (-159) polymorphism and respiratory outcomes among children in Kwazulu-Natal, Durban	Ambient air pollutants such as sulfur dioxide, NO2, NO, and PM10	*CD14* polymorphisms	Lung function
Gil-Serna al. (2018)	Description of an orthologous cluster of ochratoxin A biosynthetic genes in Aspergillus and Penicillium species. A comparative analysis	Ochratoxin (mycotoxin)	Biosynthetic genes in the main OTA-producing Aspergillus and Penicillium species (*A. steynii, A. westerdijkiae, A. niger, A. carbonarius and P. nordicum*)	Chronic interstitial nephropathy
Glass et al. (2018)	DNA variants and organophosphate neurotoxicity among emerging farmers in the Western Cape of South Africa	Pesticides (organophosphate compounds)	glutathione S-transferases (*GSTM1* and *GSTT1*), N-acetyltransferase 2 (*NAT2*), and Paraoxonase 1 (*PON1*) polymorphisms	Organophosphate neurotoxicity

^†^ G x E: genomic and environmental factors integrated in some way and evaluated as risk factors for health outcomes. G x E does not necessarily imply a statistical interaction; rather than genomic and environmental risk factors were both evaluated in the study participants, enabling the assessment of G x E interactions or related strategies.

A variety of other DNA and genomic biomarkers were also explored in relation to the effect of various exposure health risks. Aflatoxin adducts are known to be carcinogenic and mutagenic and have been associated with induction of the arginine to serine mutation in p53, and act synergistically with the hepatitis B virus to cause liver cancer (Kew, 2013). Repeated exposure to alpha-CYP pesticides appears to lead to p53 gene mutations (El Okda et al., 2017). A genotoxic impact for occupationally exposed antimony trioxide individuals was also reported with DNA damage detected in the form of increased apurinic/apyrimidic sites (El Shanawany et al., 2017). Interindividual variation in adduct levels associated with benzene and PAHs may reflect genetic susceptibility as well (Ayi-Fanou et al., 2011). One review summarized a variety of studies looking at various genotoxic biomarkers (including cytogenetic endpoints, chromosomal aberrations, etc.), DNA damage markers (including comet assay and urinary 8-hydroxydeoxyguanosine), and genomic biomarkers (including leukocyte telomere length, gene expression, etc.) (DeMarini, 2013). These markers were often able to distinguish traffic-exposed individuals from controls but only one of the 63 papers from this review was from an African-based study (DeMarini, 2013). Prenatal exposure to air pollution and HIV status of mothers appeared to lead to differential methylation in infants particularly in certain biological pathways related to metabolic processes and viral regulation (Goodrich et al., 2016). Only one study evaluated epigenome-wide DNA methylation and this study found differential methylation in genes related to growth and immune function for infants of aflatoxin-exposed mothers (Hernandez-Vargas et al., 2015). Only one study explored the possible effects on the microbiome for a particular exposure, and this report described changes in lung microbiome with high levels of black carbon particulates (Rylance et al., 2016). No genome-wide association studies (GWAS) or whole genome sequencing or RNA sequencing studies were identified in this literature review.

## Discussion

In this review, we summarize environmental health research in Africa covering the last decade, highlighting exposures unique to Africa with important health implications. Substantial progress has been made in identifying a wide range of health effects related to hazardous environmental exposures. In general, indoor and ambient air pollution studies across Africa were well characterized and health impacts are comparable to what has been described in other regions around the world. Increased industrialization, traffic, and biomass fuel burning in parts of Africa will continue to contribute to substantial air pollution. Many industrial metals contaminating the environment in parts of Africa and health effects comparable to those observed elsewhere, particularly cancer and neurological outcomes. Several reproductive outcome associations with heavy metals may be of particular interest in the African context. For example, the high levels of preeclampsia described in several African countries and the unusually high incidence of intrauterine growth retardation in Egypt may possibly be driven by toxic metal concentrations (El-Helaly et al., 2011; Ikechukwu et al., 2012; Motawei et al., 2013; El-Baz et al., 2015; Elongi Moyene et al., 2016). The acute lead poisoning for children is an urgent ongoing issue in many African countries and prevention of exposure among children is critical. A variety of pesticide studies reported reproductive, neurological, respiratory, and cancer outcomes, with one novel liver disease reporting an association with DDT (Robinson et al., 2014). The acute pesticide poisoning of adolescents (some intentional) is alarming and may reflect ease of access to these chemicals in the African continent (Balme et al., 2012; Azab et al., 2016; da Silva et al., 2016; Ssemugabo et al., 2017). Extensive mechanistic research combined with human studies over many years have allowed aflatoxin and other mycotoxins to be accurately measured and has facilitated prevention and intervention strategies. The literature on PFOS, flame retardants, phenols, e-waste, and phthalates remains extremely limited.

A variety of research gaps across multiple exposure categories were identified. The role of the immune system and inflammation and how it interacts with various exposures is an area that warrants more research. The role of endocrine disrupting chemicals in general are evolving and expanding with studies around the world and the metabolic impacts of this class of compounds, particularly for obesity, diabetes, and cardiovascular outcomes, will need to be further explored. As industrialization, urbanization, and globalization continue to impact the African continent, many emerging exposures, including PFOS, flame retardants, phenols, e-waste products, and phthalates may increase over time and will need further study in Africa.

Key susceptible/at risk populations were similar across multiple exposure categories and these include: pregnant women, children (particularly in *utero* and early childhood stages), and workers in specific occupational settings (agricultural, mining, street vendors, taxi-motorbike drivers, waste workers, etc.), and people living near urban areas who may be more highly exposed to particulate air pollutants such as benzene and PAHs. Immunocompromised individuals (people with HIV or other infections, cancer patients, etc.) may be particularly vulnerable to the effects of toxicants. The combined effects of environmental exposures and infections need to be further examined in African studies.

In general, the limited number of African studies exploring any integration or interaction of genomic and environmental factors suggests a substantial research gap. Extremely limited epigenomics and other omics applications were reported. The impact of possible transgenerational effects of some exposures by epigenomic processes has yet to be examined (Kabasenche and Skinner, 2014). The exploration of the interaction of genetic and environmental factors for disease susceptibility may enable future preventive measures. For example, the potential for agricultural workers exposed to high levels of pesticides to be screened based on genotype would be a way to help target protective measures for high risk groups and reduce disease burden (Tawfik Khattab et al., 2016). A better understanding of the regulation of biosynthetic genes related to some mycotoxins may also lead to new ways to monitor the food chain for mycotoxin contamination (Gil-Serna et al., 2018). The genetic diversity in Africa, combined with unique exposures and co-morbidities, can lead to novel G x E findings that cannot be discovered elsewhere.

### Future Directions

This review did not represent a systematic analysis of all findings reported in the literature. The purpose was to provide a broad scope of environmental health, including many complex exposure categories. Future systematic reviews could be implemented, focusing on one exposure category or a single or collection of chemicals. The greatest detail was provided for the G x E articles retrieved in this review, which, as has been noted, represented a critical research gap. Importantly, the WHO report in 2016 (Prüss-Ustün et al., 2016) stated that the current statistics related to many disease outcomes likely underestimate the true burden due to inadequate coverage in the literature, the challenge to capturing emerging risks, and the fact that many exposures take years to manifest into presentable symptoms or disease.

A number of exposures have received substantial research attention in Africa, which is encouraging, and some studies have provided unique insights that will allow further translational efforts to occur. Aflatoxin interventions and prevention efforts are a model for what could potentially be done with other exposures in a resource limited setting. Some reports were limited by exposure assessment methods (perhaps relying too heavily on questionnaires to assess exposure and health risks). Leveraging resources such as the Children’s Health Exposure Analysis Resource (CHEAR) or Human Health Exposure Analysis Resource (HHEAR) (Balshaw et al., 2017) may enable critical gains in environmental exposure measurements in biospecimens collected in African studies. Increased environmental data in coordination with genomic infrastructure such as that in the H3Africa consortium offers a strong platform for building G x E research in Africa, although collaborations should not be limited to these resources alone.

Another underrepresented area of research was geospatial methods and spatiotemporal modeling to evaluate health outcomes in African populations. The utilization of satellite data in combination with ground monitoring is challenged by inadequate coverage of ground monitoring in Africa. Involvement of data scientists and related experts is needed to leverage existing data to advance environmental health research in Africa. The application of these methods is increasingly important with ongoing and foreseeable changes in weather patterns, agriculture, industrial development, resource mining, drought, natural vegetation, and wildlife across Africa, all of which impact the habitats of vectors transmitting infectious diseases. Variability in nutrition, poverty, and infectious diseases that all impact immunity further emphasizes the importance of bolstering environmental health research capacity across the continent.

In the coming decade, we anticipate ongoing advancements in environmental health and genomics, in coordination rather than in parallel. Leveraging the resource infrastructures within Africa and the growing global collaborations that consortia and bottom up approaches are capable of, the future for G x E research in Africa is promising.

## Author Contributions

Conceived and designed the literature review: BJ, KM. Performed literature review and analysis of review results: SM. Wrote the paper: BJ, KM. Revised and approved the manuscript: BJ, KM, SM.

## Conflict of Interest

Author SM was employed by the company Vista Technology Services.

The remaining authors declare that the research was conducted in the absence of any commercial or financial relationships that could be construed as a potential conflict of interest.

## Abbreviations

BC, black carbon; CO, carbon monoxide; NO2, nitrogen dioxide; O3, ozone; PAHs, polycyclic aromatic hydrocarbons; PM, particulate matter; SO2, sulfur dioxide; VOCs, volatile organic compounds.
